# Ionic strength and hydrogen bonding effects on whey protein isolate–flaxseed gum coacervate rheology

**DOI:** 10.1002/fsn3.1504

**Published:** 2020-03-10

**Authors:** Jun Liu, Youn Young Shim, Martin J. T. Reaney

**Affiliations:** ^1^ Beijing Advanced Innovation Center for Food Nutrition and Human Health College of Food Science and Nutritional Engineering China Agricultural University Beijing China; ^2^ Department of Plant Sciences University of Saskatchewan Saskatoon SK Canada; ^3^ Prairie Tide Diversified Inc. Saskatoon SK Canada; ^4^ Department of Food Science and Engineering Guangdong Saskatchewan Oilseed Joint Laboratory Jinan University Guangzhou, Guangdong China; ^5^ Department of Integrative Biotechnology, College of Biotechnology and Bioengineering Sungkyunkwan University Suwon, Gyeonggi-do Korea

**Keywords:** complex coacervation, flaxseed gum, hydrogen bonding, ionic strength, rheological properties, whey protein isolate

## Abstract

Whey protein isolate (WPI) was mixed with anionic flaxseed (*Linum usitatissimum* L.) gum (FG), and phase transition during coacervate formation was monitored. Effects of ionic strength and hydrogen bonding on coacervation of WPI‐FG system and corresponding rheological properties were investigated. During coacervate formation, structural transitions were confirmed by both turbidimetry and confocal laser scanning microscopy. Increasing ionic strength with sodium chloride (50 mM) decreased optical density (600 nm) at pH_max_. Correspondingly, pH_c_ and pH_ϕ1_ decreased from pH 5.4 to 4.8 and from 5.0 to 4.6, respectively, while pH_ϕ2_ increased from pH 1.8 to 2.4. Sodium chloride suppressed biopolymer electrostatic interactions and reduced coacervate formation. Adding urea (100 mM) shifted pH_ϕ1_, pH_max_, and pH_ϕ2_ from 4.8, 3.8, and 1.8 to 5.0, 4.0, and 2.2, respectively, while pH_c_ was unaffected. Optical density (600 nm) at pH_max_ (0.536) was lower than that of control in the absence of urea (0.617). This confirmed the role of hydrogen bonding during coacervate formation in the biopolymer system composed of WPI and FG. Dynamic shear behavior and viscoelasticity of collected coacervates were measured, and both shear‐thinning behavior and gel‐like properties were observed. Addition of sodium chloride and urea reduced ionic strength and hydrogen bonding, resulting in decreased WPI‐FG coacervate dynamic viscosity and viscoelasticity. The disturbed charge balance contributed to a loosely packed structure of coacervates which were less affected by altered hydrogen bonding. Findings obtained here will help to predict flaxseed gum behavior in protein‐based foods.

## Graphical abstract

Effects of ionic strength and hydrogen bonding on whey protein isolate and flaxseed gum (WPI‐FG) coacervate formation and coacervate rheological properties were investigated. Structures of WPI‐FG coacervates were loosely packed due to increased ionic strength and decreased hydrogen bonding caused by NaCl and urea addition, respectively, resulting in reduced dynamic viscosity and viscoelasticity.

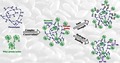

## 1 INTRODUCTION

Structural and functional properties of food biopolymers, proteins and polysaccharides, are critical in determining food product structure, texture, and stability (Ru, Wang, Lee, Ding, & Huang, 2012). Food products are usually complex matrixes with different combinations of biopolymers (Schmitt & Turgeon, 2011). During food processing, biopolymers might act as synergists or antagonists and are potentially cosoluble, incompatible, or form complex coacervates (Doublier, Garnier, Renard, & Sanchez, 2000; de Kruif & Tuinier, 2001). Complex coacervates composed of protein and polysaccharides are widely studied as their structure and physical properties can affect performance and utility of foods, pharmaceuticals, and cosmetics (Turgeon, Schmitt, & Sanchez, 2007). Protein–polysaccharide coacervate solubility is determined by electrostatic interactions, hydrogen bonding, and/or hydrophobic interactions (Espinosa‐Andrews, Baez‐Gonzalez, Cruz‐Sosa, & Vernon‐Carter, 2007). Thus, knowledge of biopolymer interactions is helpful as a basis to engineer the structural and functional properties of food products.

Properties of protein–polysaccharide coacervates make them useful for different applications, such as nutrient encapsulation, separation and/or recovery of proteins, enzyme immobilization, emulsification, gelatinization, and/or foam stabilization (Zhao et al., 2014). Many parameters could determine the formation of complex coacervates between biopolymers through affecting biopolymer interaction strength and biopolymer system entropy (Ball et al., 2002; de Kruif, Weinbreck, & Vries, 2004; Ou & Muthukumar, 2006). Moreover, rheological properties of protein–polysaccharide coacervates are also important in determining their potential utilization as fat replacers, meat analogues, coatings, edible films, etc. (Liu, Shim, Shen, Wang, & Reaney, 2017; Liu, Shim, Tse, Wang, & Reaney, 2018). Texture and overall sensory acceptability of food products are improved by designing protein–polysaccharide coacervates with preferred rheological properties (Espinosa‐Andrews, Sandoval‐Castilla, Vázquez‐Torres, Vernon‐Carter, & Lobato‐Calleros, 2010; Huang, Xiao, Wang, & Qiu, 2015). For food development and quality control it is critically important to understand protein–polysaccharide coacervate structural and rheological properties as functions of environment.

In the current study, gum was extracted from whole flaxseed and coacervates were formed with whey protein isolate (WPI). Whey protein isolate is produced by precipitation of protein, largely casein, from milk at pH 4.6 and 20°C (Weinbreck, Nieuwenhuijse, Robijn, & Kruif, 2004). Beta‐lactoglobulin, with an isoelectric point (IEP) of 5.2, and α‐lactalbumin, with an IEP of 4.1, are primary components of WPI and have excellent nutritional properties. In food formulations, WPI contributes versatile functional properties, enabling emulsification, gelation, and foaming (Turgeon & Beaulieu, 2001). Flaxseed gum (FG) constitutes about 8% of dry flaxseed mass and is recovered mainly as an extract of flaxseed hull that is released when whole or milled seed is soaked in water (Ziolkovska, 2012). Flaxseed gum solutions are separable to yield two distinct polysaccharide fractions: a neutral arabinoxylan of 1.2 × 10^6^ Da which constitutes 75% of FG mass (Qian, Cui, Wu, & Goff, 2012); and an acidic rhamnogalacturonan‐I (RG‐I) polysaccharide (Qian, Cui, Nikiforuk, & Goff, 2012). The acidic fraction is further separable into two fractions with molecular weights of 6.5 × 10^5^ Da and 1.7 × 10^4^ Da, constituting 3.75% and 21.25% of the mass of FG, respectively (Qian, Cui, Wu, et al., 2012). The neutral fraction of FG was reported to be largely free of uronic acid. Although, uronic acid was identified to account for 1.8% of the mass of a neutral fraction of 1.47 × 10^6^ Da isolated from FG (Qian, Cui, Wu, et al., 2012). Flaxseed gum solutions exhibit useful rheological properties that enable them to be considered for use in emulsification, gelation, foam formation, and foam stabilization (Singh, Mridula, Rehal, & Barnwal, 2011). Flaxseed gum solution functional properties have led to studies of FG utilization in food products of dairy dessert, sausage, salad dressing, carrot juice, etc. (Stewart & Mazza, 2000; Zhou, Meng, Li, Ma, & Dai, 2010). Moreover, FG has been reported to show health benefits when consumed as dietary fiber. Recently, Health Canada has approved a claim that consuming 40 g of flaxseed daily reduces cholesterol and this finding is consistent with the consumption of a diet high in soluble dietary fiber. Other potential positive impacts of consuming such a diet include reduced risk of diabetes, coronary artery diseases, colon and rectal cancers, and prevention of obesity (Cunnane et al., 1993; Singh et al., 2011; Thakur, Mitra, Pal, & Rousseau, 2009).

Previously, WPI‐FG coacervates were formed over a wide range of biopolymer mixing ratios (*R* = [WPI]/[FG], 1:4–15:1, w/w) and pHs (6.0–1.4). At pH 3.8, WPI‐FG coacervate formation reached a maximum with *R* of 2:1 (w/w). Accordingly, the WPI‐FG coacervates formed at this ratio demonstrated the highest dynamic viscosity and viscoelasticity. Electroneutrality favored coacervate formation, resulting in more compact structures with improved apparent viscosity and viscoelasticity (Liu et al., 2017). In this research, we investigated the contributions of ionic strength and hydrogen bonding on WPI‐FG coacervate structure and rheological properties. Phase transitions during coacervate formation were monitored, and effects of ionic strength and hydrogen bonding on WPI‐FG coacervate rheology were evaluated with the addition of NaCl and urea, respectively. Findings from these studies will provide basic information needed to predict FG behavior in protein‐based food products. Flexible mechanical and structural properties of WPI‐FG coacervates offer great potential for targeted food applications with desired contributions to food properties.

## 2 MATERIALS AND METHODS

### 2.1 Materials

Flaxseed (*Linum usitatissimum* L. var., CDC Bethune) used in this study was provided as a generous gift by Drs. G. Rowland and H. Booker (Saskatchewan Crop Development Centre, Saskatoon, SK, Canada). Whey protein isolate (*Bi*PRO) was purchased from Davisco Foods International., Inc. Urea and rhodamine B (fluorescence grade) were obtained from Sigma‐Aldrich. NaCl was purchased from EMD Millipore Corporation. Anhydrous ethanol was purchased from Commercial Alcohols Inc. HCl (37%, w/w) was obtained from Fisher Scientific Company. Deionized RO water was produced by Milli‐Q system. All other reagents were of analytical grade and used as received.

### 2.2 Gum preparation from whole flaxseed

Surface dust on flaxseed was removed by rinsing with tap water. Then, extraction was performed at a flaxseed to deionized RO water ratio of 10:1 (w/w) under gentle stirring (300 rpm). Extraction temperature was maintained at 60°C for 24 hr (Wang, Wang, Li, Xue, & Mao, 2009). Extracts were filtered through cheesecloth to separate soaked flaxseed. After separating the seed, FG extracts were centrifuged at 12,700 *g* for 20 min (4°C) to remove insoluble particles. Then, supernatants were decanted, and FG fractions were precipitated with ethanol (1:1, v/v). The precipitates were centrifuged, decanted, resuspended in water, freeze‐dried, and subsequently used as FG samples.

### 2.3 Stock solution preparation

Accurately weighed WPI and FG were dispersed in deionized RO water and stirred (300 rpm) at RT for 2 hr. To ensure full hydration, each biopolymer dispersion was then held at 4°C overnight to prepare stock solutions (0.05%, w/w) for coacervation. For rheological measurements, large amounts of WPI‐FG coacervates were needed. Therefore, the concentration of stock solution of WPI and FG was 1.0% (w/w), respectively, and was prepared following the same procedures as described.

### 2.4 WPI coacervate with FG

At a total biopolymer concentration (*C*_T_) of 0.05% (w/w), WPIs were mixed with FG with *R* = 1:1 (w/w). The biopolymer mixture was titrated with HCl, and coacervate formation was observed using turbidimetric analysis. Glucono‐δ‐lactone solution (0.005%, w/w), an internal acidifier, was added until the pH was lowered to 4.4. Then, the pH was further decreased following the gradient of pH 4.4–3.6, pH 3.6–2.8, pH 2.8–2.0, and pH 2.0–1.4 with the dropwise addition of 0.05 M, 0.5 M, 1.0 M, and 2.0 M HCl solution, respectively (Weinbreck, Vries, Schrooyen, & Kruif, 2003). The increased molarity of HCl with decreased pH is required to mitigate dilution during titration. Urea (0, 50, and 100 mM) and NaCl (0, 25, and 50 mM) were added to disrupt hydrogen and ionic bond strength, respectively, and, thereby, determine bond effects on coacervate formation. WPI‐FG coacervates prepared at *C*_T_ of 1.0% (w/w) as a function of NaCl (0–200 mM) or urea (0–200 mM) were used for rheological measurements. HCl solution (2.0 M) was used to adjust the pH of biopolymer mixtures. Coacervates were collected by centrifugation at 3,000 *g* for 20 min (25°C).

### 2.5 Turbidity measurement

During acid titration, WPI‐FG mixture optical density at 600 nm (OD_600_) was measured in the presence/absence of NaCl or urea. Turbidity (*τ*, cm^–1^) of WPI‐FG mixture during the acid titration was defined as follows:

(1) $$\tau =-\left(\frac{1}{L}\right)\ln\left(\frac{I}{I_{0}}\right)$$

where *L* is the path length of light (1 cm), *I* is the radiation intensity in the presence of sample, and *I*_0_ is the light intensity in the presence of distilled water.

WPI‐FG mixture turbidity was plotted versus solution pH and used to determine transition points. Phase transition point pH_c_ was defined as where the turbidity was slightly increased due to the soluble “primary” coacervates. The intersection of two curve tangents was regarded as pH_φ1_, and pH_φ2_, indicating the formation and totally dissociation of insoluble WPI‐FG coacervates, respectively (Weinbreck, Nieuwenhuijse, Robijn, & Kruif, 2003). The pH where the highest OD_600_ was demarcated as pH_max_ was reached, creating the maximum interactions between WPI and FG in solution to form coacervates.

### 2.6 Confocal laser scanning microscopy

Effects of NaCl (0–50 mM) and urea (0–100 mM) on structural properties of WPI‐FG coacervates (*R* = 1:1, *C*_T_ = 0.05%, w/w; pH 3.4) were monitored. Before formation of coacervates between WPI and FG (20.0 ml), WPI was stained by 0.01% (w/w) Rhodamine B solution (10.0 µl) with stirring for 1 hr and then mixed with FG dispersions. Images of stained samples were collected by confocal laser‐scanning fluorescence microscope (Zeiss LSM‐510) and further processed by LSM 510 image analysis software (Version 3.2).

### 2.7 Rheological measurements

Rheological properties of WPI‐FG coacervates were analyzed AR2000ex rheometer (cone plate geometry of 2°, 40 mm diameter, and 0.054 mm gap) coupled with Peltier temperature control system (TA Instruments Ltd.). Solvent evaporation and other environmental interference during rheological testing were mitigated using a solvent trap. After 2 min equilibration, continuous shear (0.1–100 s^−1^) was applied onto the coacervates (*R* = 1:1, *C*_T_ = 1.0%, w/w; pH 3.4) for the construction of experimental flow curves. Oscillatory tests were conducted to investigate the viscoelasticity of WPI‐FG coacervates. A 0.1% strain amplitude was selected for all frequency sweep tests based on strain sweep measurements at 1.0 Hz frequency. The loaded samples of coacervates were tested over 0.628–628 rad/s, and TA Rheology Advantage Data Analysis Software (Version 5.4.7) was used for analysis.

## 3 RESULTS AND DISCUSSION

For protein–polysaccharide mixture of natural food origin, coacervates were formed when electrostatic interactions and non‐Coulombic forces of hydrogen bonding, hydrophobic interactions, and steric forces were favored (Xiong et al., 2017). Such coacervates have low toxicity, are biodegradable, and potentially combine the functional properties of both biopolymers (Laneuville, Paquin, & Turgeon, 2005). Accordingly, protein–polysaccharide coacervates are used foods where their rheological properties determine functionality. The magnitude and range of enthalpic factors can influence coacervate formation, microstructure, stability, and rheological properties (Chung & McClements, 2015). Viscoelasticity of coacervates formed between gum Arabic (GA) and whey proteins increased with improved electrostatic interaction as a function of acidity (Weinbreck, Wientjes, Nieuwenhuijse, Robijn, & Kruif, 2004). Addition of NaCl might decrease enthalpic contributions by shielding charges of pectin and bovine serum albumin (BSA), thereby, conferring reduced *G*′ and *G*″ values (Ru et al., 2012). However, at pH 3.0, O‐carboxymethyl chitosan (OCC) formed maximum coacervates with GA while the coacervates of the same system formed at pH 6.0 had the highest viscosity and viscoelasticity. These observations indicate that factors beyond electrostatic interactions, such as hydrogen bonding, might contribute to the rheological properties of OCC‐GA coacervates (Huang et al., 2015). Similarly, agar–whey protein coacervates formed in citrate buffer produced higher modulus values than coacervates formed in water (Rocha, Souza, Magalhães, Andrade, & Gonçalves, 2014). In this study, the contributions of electrostatic interaction and hydrogen bonding to WPI‐FG coacervate formation and rheological properties were studied to help resolve the above discrepancies.

### 3.1 Effects of NaCl on WPI‐FG coacervation

**Figure 1:**
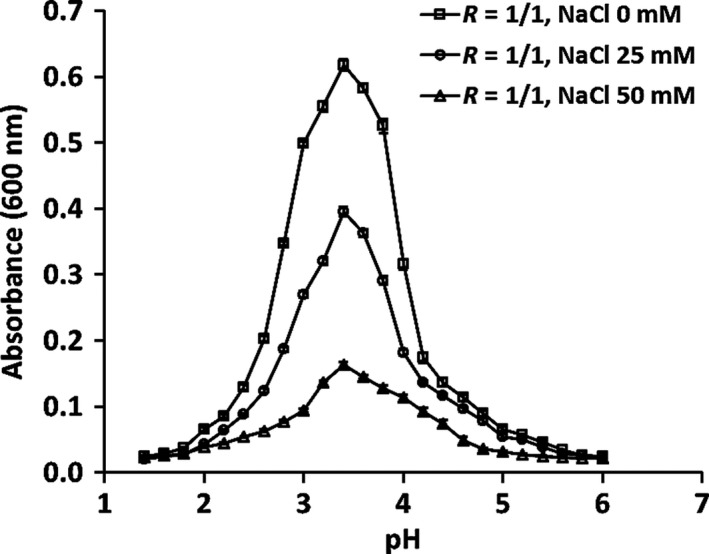
Effects of NaCl concentration (0–50 mM) on WPI‐FG coacervation within pH 6.0–1.4 at *R* = 1:1 (w/w)

Electrostatic interactions were first identified as a primary force guiding the formation of coacervates of gelatin and acacia gum (Tiebackx, 1911). Since this pioneering work coacervate formation has been studied for many protein and polysaccharide systems, including FG and BSA (Liu, Shim, Wang, & Reaney, 2015). Soluble and insoluble FG‐BSA coacervate formations are determined by solution pH and ionic strength as these factors can affect the ionization of amino and carboxylic groups on biopolymer structures and interactions between charge groups in solution. The contribution of electrostatic interactions to WPI‐FG coacervate formation was investigated with the addition of NaCl. As shown in acid titration curve (Figure 1), pH_c_ decreased from pH 5.4 to 4.8 with increasing NaCl concentration from 0 to 50 mM, and similarly, pH_φ1_ decreased from pH 5.0 to 4.6.

At the phase transition points of pH_c_ and pH_φ1_, OD_600_ decreased with increased NaCl concentration, indicating decreased formation of soluble WPI‐FG complexes. In the reaction mixture, dissolved ions of NaCl can compete with negatively charged groups on FG polysaccharides and positively charged groups on whey proteins. Thus, electrostatic interaction strength between FG and WPI molecules was reduced due to the masked biopolymer charges. With the addition of NaCl, pH_c_ and pH_φ1_ were decreased to give more positive charges on molecules of WPI (Girard, Turgeon, & Gauthier, 2002). However, for gelatin‐agar mixtures, pH_c_ was constant with NaCl concentrations below 200 mM while pH_φ1_ moved to a lower pH with salt concentrations above 50 mM (Singh et al., 2007). This could be due to the increased gelatin solubility by enhancing molecular coiling effects at a critical NaCl concentration lower than 200 mM (Schmitt, Sanchez, Thomas, & Hardy, 1999). Higher concentrations of dissolved protein increase total positive charges and thus stabilize pH_c_ during acid titration. During coacervate formation between β‐lactoglobulin and pectin, an increased pH_φ1_ and independent turbidity of biopolymer system were observed with NaCl concentration lower than 100 mM and 800 mM, respectively. Aggregation of β‐lactoglobulin molecules during acid titration was responsible for an increase in pH_φ1_ and constant solution turbidity (Wang, Lee, Wang, & Huang, 2007).

**Figure 2:**
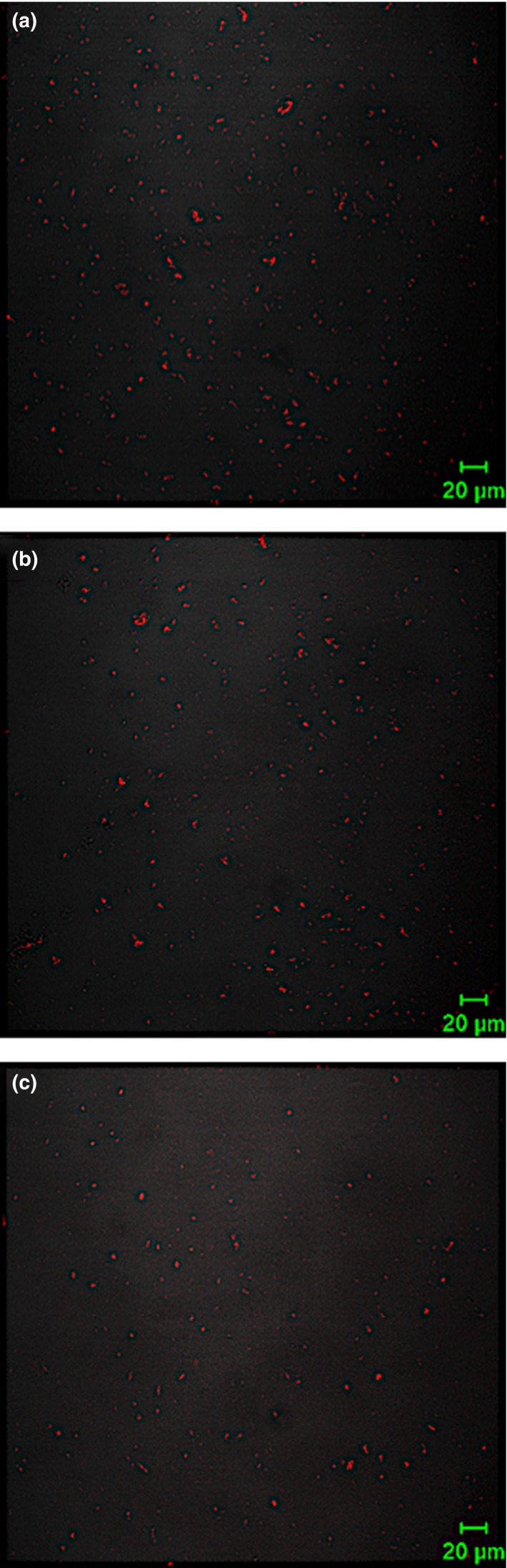
Effects of NaCl addition on microstructure of WPI‐FG coacervates formed at pH 3.4 (*R* = 1:1, w/w): (a) blank control; (b) NaCl, 25 mM; (c) NaCl, 50 mM

As shown in Figure 1, WPI‐FG mixture pH_max_ was independent of NaCl concentration (0–50 mM) though OD_600_ at pH_max_ decreased with addition of salt. This phenomenon could be ascribed to WPI protein composition. Whey protein isolate proteins have an isoelectric point (IEP) that compensates for decreased positive charge density on protein molecules caused by NaCl charge screening effects (Weinbreck, Vries, et al., 2003). However, pH_max_ decreased from 4.0 to 3.6 for FG‐BSA coacervates when NaCl was added at 100 mM (Liu et al., 2015). Down‐shifted pH favored protein lysine ionization (−NH_2_) and increased positive charge density on BSA molecules. This compensated for charge screening by salt and supported complex formation between FG and BSA (Girard et al., 2002). Solution OD_600_ of WPI‐FG decreased from 0.617 to 0.163 at pH_max_ of 3.4 with increased NaCl concentration from 0 to 50 mM, respectively (Figure 1). This provided evidence that insoluble coacervate formation between WPI and FG was suppressed by salt addition. Confocal laser scanning microscopy observation confirmed suppression of WPI‐FG coacervate formation by NaCl (0–50 mM) (Figure 2). With the addition of NaCl to 50 mM (Figure 2c), both the size and number of WPI‐FG coacervate particles were decreased as compared with that formed in the absence of NaCl (Figure 2a). Due to the screening effects of dissolved Na^+^ and Cl^−^ on amino and carboxylic groups, attractive electrostatic interactions were reduced resulting in weakened aggregation of soluble complexes.

With increasing NaCl concentration (0–50 mM), pH_φ2_ of WPI‐FG increased from pH 1.8 to 2.4. Thus, the difference between pH_φ1_ and pH_φ2_ was reduced. Total dissociation of insoluble WPI‐FG coacervates occurred at higher pH with the addition of salt (NaCl) than that of control. This can be ascribed to charge screening effects of dissolved Na^+^ and Cl^−^ ions on WPI and FG biopolymers where repulsive electrostatic interactions dominated at higher pH. Similar effects of NaCl on turbidity titration curves were reported for WPI‐GA biopolymer system. With the increasing of NaCl concentration above 20.3 mM, both pH_c_ and pH_φ1_ decreased while pH_φ2_ increased. Biopolymer charge screening by added ions was responsible for decreased separation between pH_φ1_ and pH_φ2_ during WPI‐GA coacervate formation (Weinbreck, Vries, et al., 2003). Xiong et al. (2017) also observed a narrowed region for ovalbumin–carboxymethylcellulose coacervate formation between pH_φ1_ and pH_φ2_ with increasing ionic strength (NaCl concentration from 0 to 400 mM). At higher ionic strength, the Debye length (*R*_d_ = 0.3/$\sqrt{C_{\text{NaCl}}}$, nm) is less than ovalbumin molecular radius (*R*_pro_), resulting in total screened long‐range repulsive interactions and weakened short‐range attractive interactions (Seyrek, Dubin, Tribet, & Gamble, 2003), thus ions suppressed coacervate formation.

### 3.2 Effects of urea on WPI‐FG coacervation

**Figure 3:**
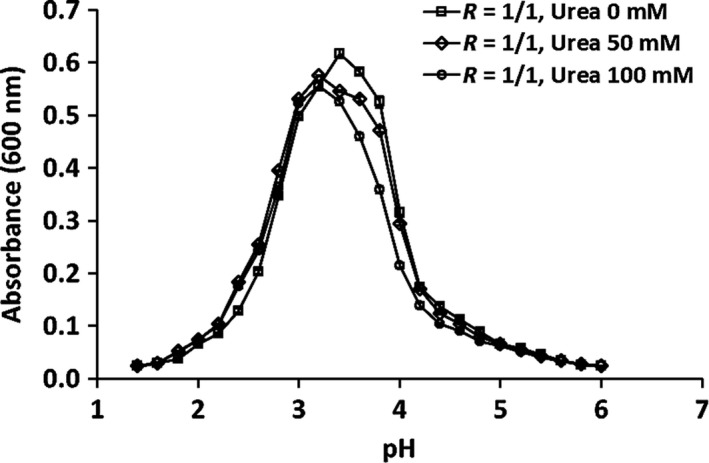
Effects of urea concentration (0–100 mM) on WPI‐FG coacervation within pH 6.0–1.4 at *R* = 1:1 (w/w)

It is well accepted that electrostatic interactions contribute to protein–polysaccharide coacervate formation. However, electrostatic interactions are not always essential to form protein–polysaccharide coacervates. Intra‐ and intermolecular hydrogen bonding and hydrophobic interactions can also affect phase separation during protein–polysaccharides interactions (Wang et al., 2015). Antonov and Gonçalves (1999) investigated the influence of temperature on phase separation behavior of gelatin‐κ‐carrageenan (KC) mixtures. During cooling from 50 to 20°C, turbidity of gelatin‐KC mixture was not changed at a high mixing ratio. It was concluded that both hydrogen bonding and hydrophobic interactions were insignificant for insoluble gelatin‐KC coacervate formation. However, Liu et al. (2010) found that pea protein isolate‐GA complex formation was significantly improved as the temperature decreased from 23 to 6°C. While with increasing of temperature from 23 to 60°C, the stability of insoluble coacervates was improved. At lower temperatures, hydrogen bonding was favored as a secondary force for coacervate formation, whereas biopolymer conformation was changed by heat treatment, exposing interaction sites and making the hydrophobic interactions more prevalent. In this study, contributions of hydrogen bonding to WPI‐FG coacervate formation (*C*_T_ = 0.05% and *R* = 1:1, w/w) and rheological properties were investigated in the presence of the denaturant urea (0–100 mM), which can change the aqueous phase structure around biopolymer molecules. In the presence of urea, biopolymer hydrophobic group solubility increases, resulting in disrupted hydrogen bonding (Liu, Low, & Nickerson, 2009). Titration of WPI‐FG mixtures after addition of urea (50 and 100 mM) produced turbidity curves with a similar pattern to urea free controls. Increased urea concentration moved turbidity curve peaks to a more acidic pH (pH 3.2) than controls (pH 3.4; Figure 3).

Consistently, with the addition of urea (100 mM), OD_600_ of the reaction mixture at pH_max_ reached 0.556 which was lower than that of control 0.617, indicating suppressed WPI‐FG coacervate formation. This was probably caused by weakened hydrogen bonds. The influence of urea addition on OD_600_ at pH_max_ was smaller than that of NaCl at the same concentration. This was consistent with previous research on coacervate formation which showed that electrostatic interactions were the primary interaction for BSA‐FG coacervate stabilization while hydrogen bonding had less effect (Liu et al., 2015).

**Figure 4:**
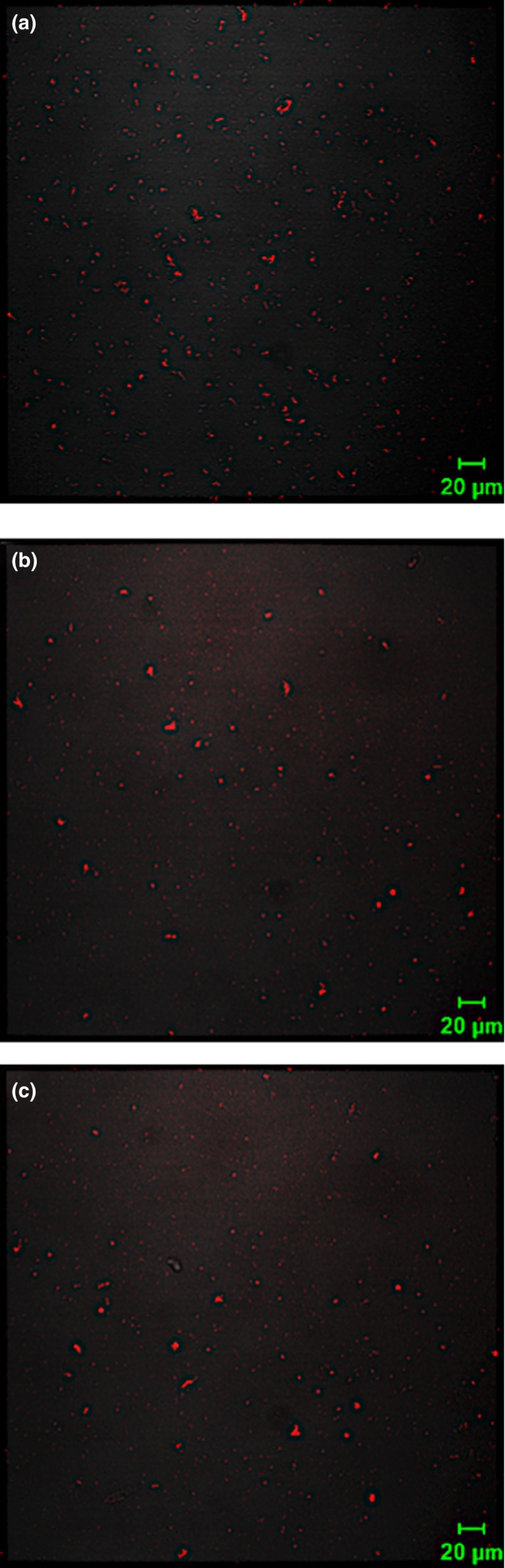
Effects of urea addition on microstructure of WPI‐FG coacervates formed at pH 3.4 (*R* = 1:1, w/w): (a) blank control; (b) urea, 50 mM; (c) urea, 100 mM

Turbidity changes during titration were consistent with confocal laser scanning microscopy observations (Figure 4). The quantity of WPI‐FG coacervates observed when urea concentration increased from 0 to 50 mM was slightly reduced (Figure 4a,b). Urea suppression of WPI‐FG coacervate formation confirmed the decrease in hydrogen bond formation between biopolymer molecules. When urea concentration was 100 mM (Figure 4c), no additional changes of size and number of WPI‐FG coacervates were observed by confocal laser scanning microscopy. During acidic titration (Figure 3), critical values of pH_φ1_, pH_max_, and pH_φ2_ were shifted to 4.8, 3.2, and 1.6 in the presence of 50 mM urea as compared to the urea free control (5.0, 3.6, and 1.8). The pH shifts also confirmed the contribution of hydrogen bonding to WPI‐FG coacervates while electrostatic attractive interactions were primarily responsible for the formation of insoluble coacervates. However, no changes on phase transition points were observed with further increasing urea concentration to 100 mM. Similar results were reported by Aryee and Nickerson (2012) for the coacervates of lentil protein isolates and GA. Phase transition points of pH_c_, pH_φ1_, and pH_max_ were shifted to lower pH with the addition of 100 mM urea. In this study, pH_c_ was independent of urea concentration (0–100 mM). For a β‐lactoglobulin coacervate with highly methylated pectin, no variation in pH_c_ was induced by urea below 150 mM, but pH_c_ was lower by 0.5 pH units (4.5) with the addition of 110 mM urea for coacervates formed with less methylated pectin. This indicated that hydrogen bonding played a minor role during formation of complex coacervates of β‐lactoglobulin and highly methylated pectin (Girard et al., 2002).

Consistently, with urea concentration below 150 mM, pH_c_ value was not affected for BSA‐FG mixtures. However, pH_φ1_, pH_max,_ and pH_φ2_ were shifted from 5.0, 4.0, and 2.2 to 4.8, 3.8, and 1.8, respectively, and OD_600_ at pH_max_ was decreased (0.818 to 0.664) (Liu et al., 2015). Data support the hypothesis that hydrogen bonding stabilizes WPI‐FG coacervates formed between with electrostatic attractive interactions as the primary driving force.

### 3.3 Rheological properties of WPI‐FG coacervates

#### 3.3.1 Dynamic shear behavior

**Figure 5:**
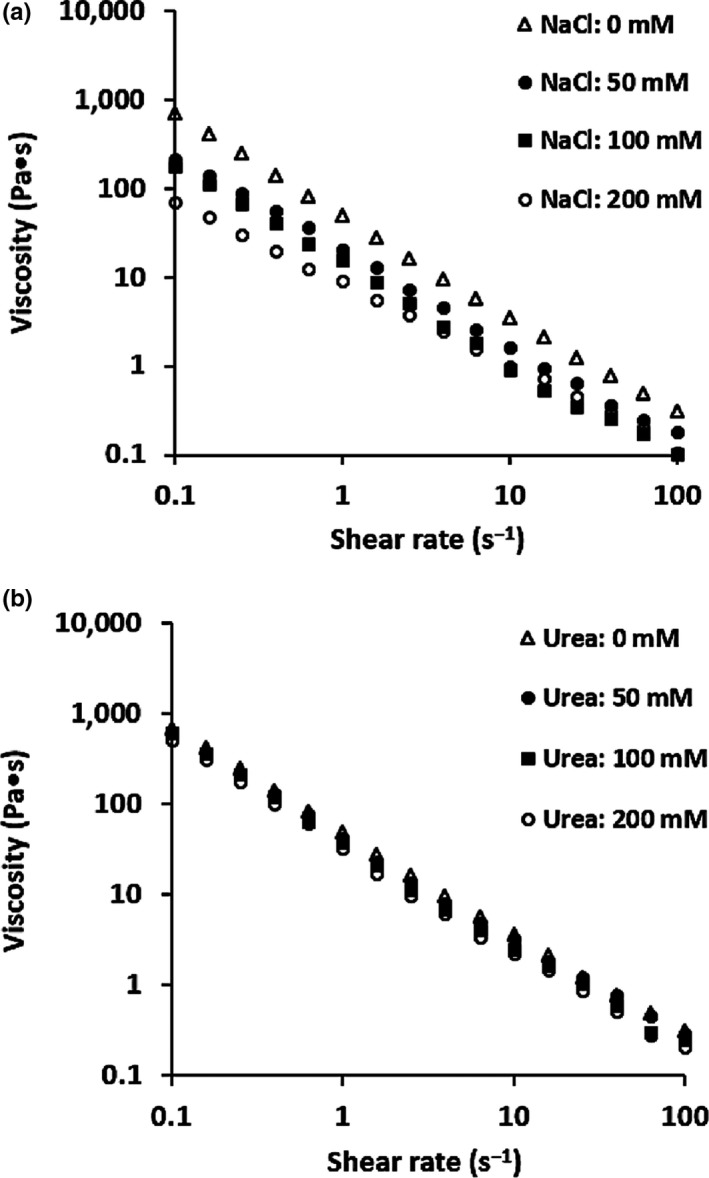
Effects of NaCl concentration (a) and urea concentration (b) on dynamic viscosity of WPI‐FG coacervates

Coacervates formed between WPI and FG at pH 3.4 in the presence of NaCl (0–200 mM) were used as representatives (*C*_T_ = 1.0%, w/w). As shown in Figure 5a, the dynamic viscosity of coacervates decreased with increasing shear rate, indicating shear‐thinning behavior. Flaxseed gum polysaccharide chains in WPI‐FG coacervates possibly contributed much of the observed unique dynamic shear behavior (Liu et al., 2016). Consistently, NaCl was reported to decrease intramolecular charge repulsion between FG polysaccharide chains, thus reducing number of junction zones and consequently FG gel strength (Chen, Xu, & Wang, 2006).

Similar effects of NaCl (0–100 mM) on dynamic shear behavior were observed on coacervates formed between sodium caseinate and pectin (Weinbreck, Wientjes, et al., 2004). Structural breakdown or rearrangement of the WPI‐FG coacervates under applied shearing force might contribute to shear‐thinning behavior (Wee et al., 2014). Flaxseed gum polysaccharide chains formed the highest strength of electrostatic attractive forces with WPI molecules at pH 3.4 with no charge screening effects caused by dissolved salt ions. Low salt environment resulted in a tightly packed WPI‐FG coacervate structure and higher apparent viscosity (Stone, Teymurova, & Nickerson, 2014). Findings here were in agreement with our previous results of a WPI‐FG mixture (*R* = 1:1, w/w) zeta potential. Electric neutrality of the WPI‐FG system occurred at the pH_max_ of 3.4, leading to the highest biopolymer mixture solution OD_600_ (0.783). However, increased NaCl concentration in WPI‐FG mixture suppressed coacervate formation by screening charges with dissolved Na^+^ and Cl^−^ ions and led to reduced WPI‐FG coacervate formation and a loosely packed coacervate structure. Similar results were also reported by Espinosa‐Andrews et al. (2013) with coacervate viscosity being related to attractive electrostatic interactions strength which was, in turn, affected by biopolymer solution ionic strength. OCC‐GA coacervates collected at pH 3.0 are typical shear thinning at shear rate lower than 60 s^−1^. However, further increment of shear rate 60–100 s^−1^ caused structural rearrangement of OCC‐GA coacervates, leading to increased viscosity. When beyond the critical shear rate of 60 s^−1^, the applied shear might cause reconstruction of destroyed coacervate structure (Huang et al., 2015). However, Chung and McClements (2015) observed increased apparent viscosity of sodium caseinate–pectin coacervates when NaCl concentration was above 100 mM. Hydrogel particles comprised of sodium caseinate–pectin coacervates and water were formed with casein‐rich particles coated with pectin molecules. The increased apparent viscosity could be ascribed to increased porosity and nonsphericity, thus improved effective volume fraction of caseinate–pectin coacervates formed at NaCl concentrations higher than 100 mM.

Coacervates formed from WPI‐FG biopolymer mixtures were also responsive to urea as it decreases hydrogen bonding (Ye, 2008). Here, effects of urea concentration (0–200 mM) on shear flow properties of WPI‐FG coacervates (pH 3.4, *C*_T_ = 1.0%, and *R* = 1:1, w/w) were investigated (Figure 5b). Typical shear‐thinning flow behavior was observed for WPI‐FG coacervates formed in the presence of urea. Electroneutrality was achieved at pH 3.4 for the biopolymer mixture which contributed to the highest electrostatic interaction strength (Yang, Chen, & Chang, 1998). Correspondingly, WPI‐FG coacervates collected under above pH condition (3.4) demonstrated the highest resistance to shear (Espinosa‐Andrews et al., 2013). At the same pH, apparent viscosity of coacervates was decreased to 32.67 Pa・s (1.0 s^−1^) in the presence of 200 mM urea. Urea suppressed hydrogen bonding that played a secondary role in stabilizing the WPI‐FG coacervates. Even though the highest strength of electrostatic interactions was achieved at pH 3.4, formation of WPI‐FG coacervates was reduced in the presence of urea. Apparent viscosity of a WPI‐FG coacervate produced with 200 mM urea was lower than that of urea free controls. The lower viscosity was also induced through suppressed hydrogen bonding (Schmitt et al., 1999).

### 3.4 Viscoelastic properties

**Figure 6:**
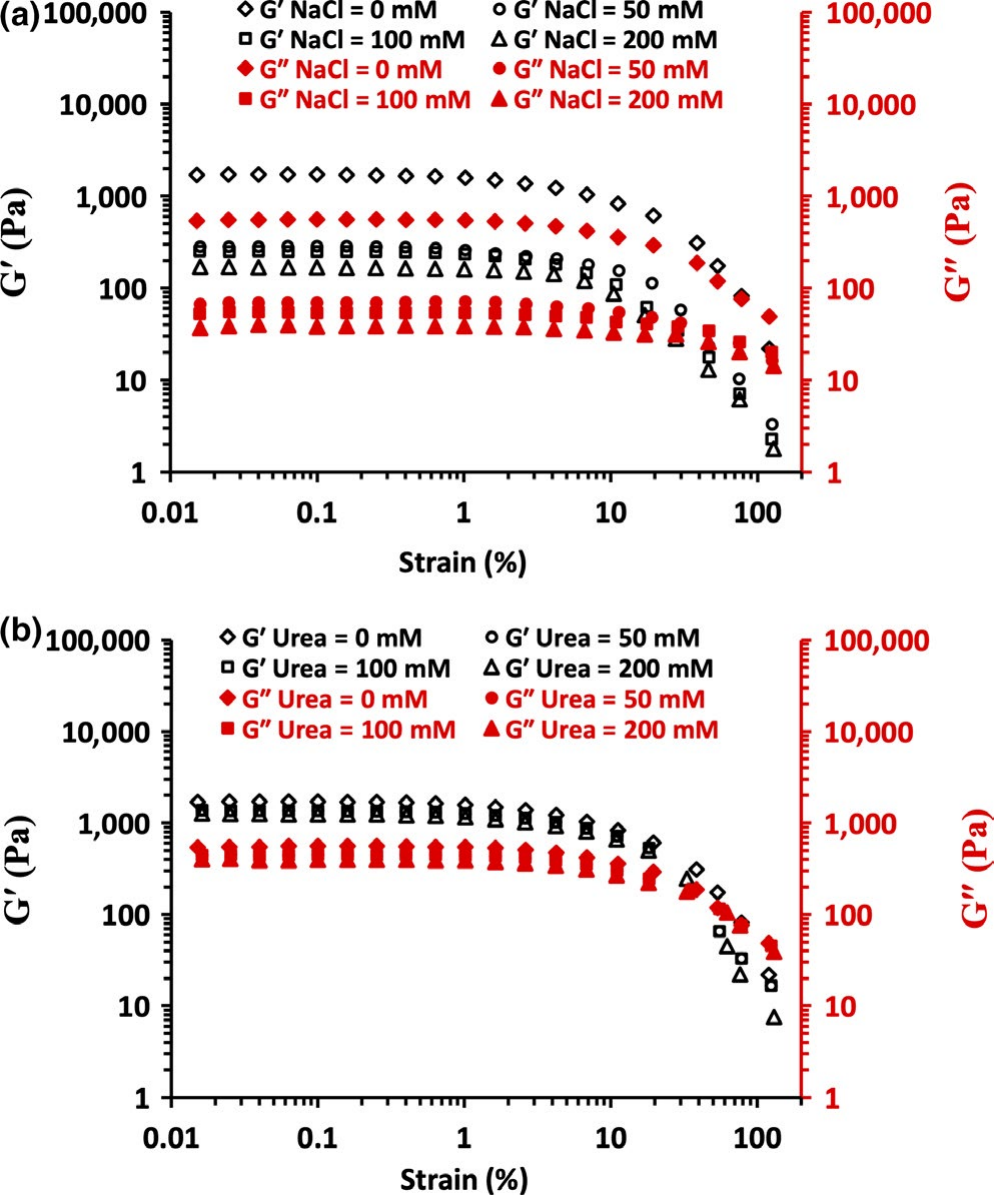
Effects of NaCl concentration (a) and urea concentration (b) on *G*′ and *G*″ of WPI‐FG coacervates as determined by strain sweep at 6.28 rad/s

**Figure 7:**
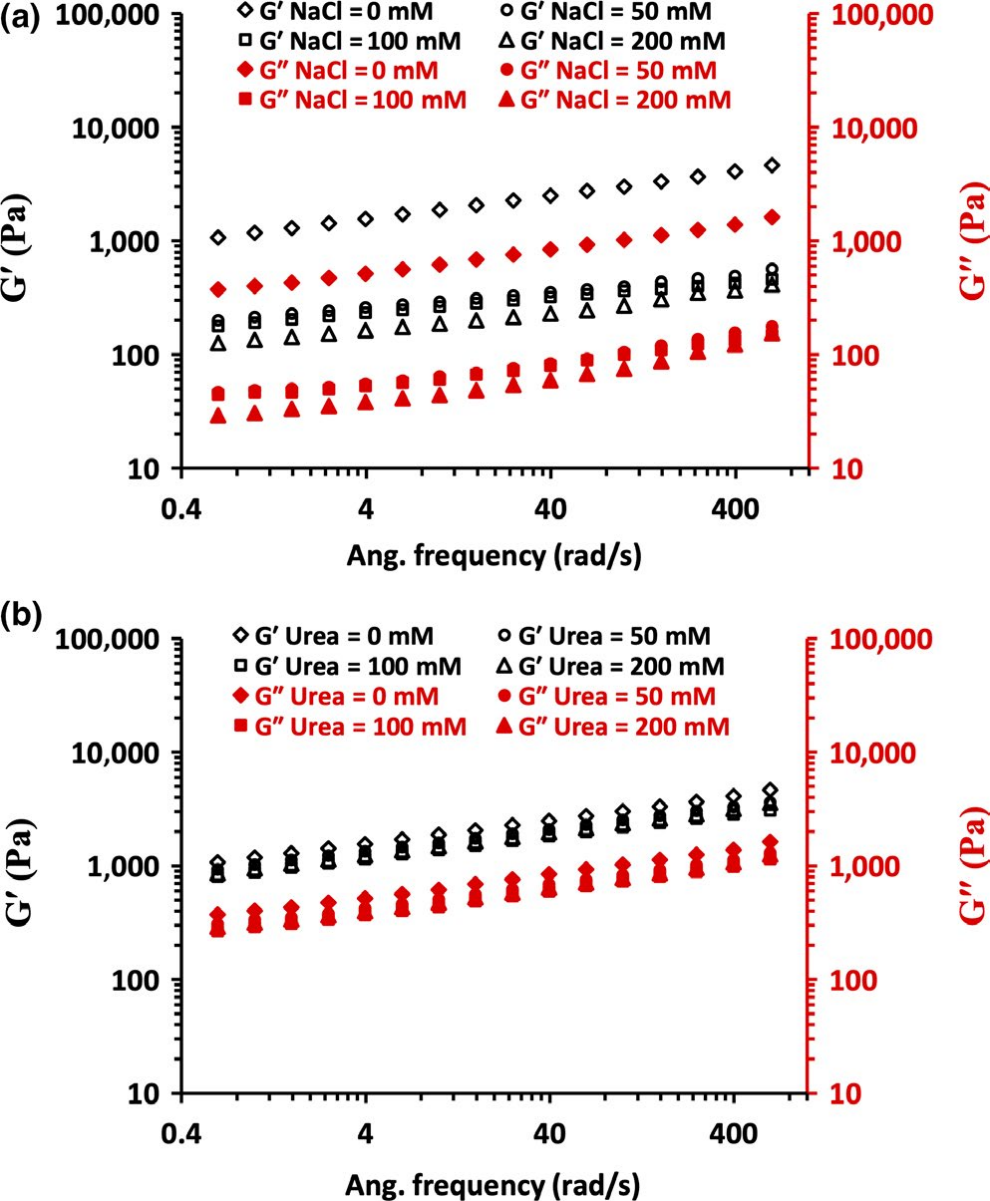
Effects of NaCl concentration (a) and urea concentration (b) on *G*′ and *G*″ of WPI‐FG coacervates as determined by frequency sweep tests at 0.1% strain amplitude

As shown in Figure 6a,b, 0.1% strain amplitude was chosen for frequency sweep testes on WPI‐FG coacervates. Frequency sweep curves as a function of either NaCl or urea concentration on WPI‐FG coacervates are shown in Figure 7a,b. Over the frequency range tested, *G*′ of WPI‐FG coacervates collected at pH 3.4 (*R* = 1:1, w/w) was higher than that of *G*″, indicating gel‐like properties due to the interconnected biopolymer chains in the coacervate structure. With increasing NaCl concentration (0–200 mM), both dynamic *G*′ and *G*″ decreased, indicating the deformation of weaker gel‐like network structures in the presence of salt (Figure 7a).

Charge screening effects of NaCl on WPI‐FG system biopolymer molecules induced a loosely packed structure of coacervates while increased total bound water. These results were consistent with variation in turbidity and confocal laser scanning microscopy observations as affected by NaCl. Many studies have reported that electrostatic screening effects of NaCl could weaken biopolymer coacervate rheological properties. For a biopolymer system composed of sodium caseinate–gum tragacanth (*Astragalus rahensis*), the strongest viscoelasticity occurred at pH 4.04 while either increased or decreased pH induced a decrement of *G*′ and *G*″ (Gorji, Gorji, Mohammadifar, & Zargaraan, 2014). Higher ionic strength (20–400 mM) decreased viscoelastic properties of ovalbumin–carboxymethylcellulose coacervates through shielded biopolymer charge, resulting in a loosely packed coacervate structure (Xiong et al., 2017). However, a few exceptions have been reported. Wang, Wang, and Heuzey (2016) found that viscoelasticity of gelatin type B‐chitosan coacervates was enhanced in the presence of NaCl due to reduced water content in the coacervate gel structure. For coacervates formed between β‐lactoglobulin and pectin, *G*′ increased within NaCl concentration range of 0.01–0.21 mol/L. However, further increases in NaCl concentration caused a large decrease in *G*′ (Wang et al., 2007), and a great increase in *G*″ was observed for N,O‐carboxymethyl chitosan (NOCC)‐GA coacervates when NaCl reached 250 mmol/L. These findings can be interpreted as an effect of lowered water content while more compact coacervate structures occurred with higher NaCl concentration. Thus, coacervates produced in higher NaCl were more resistant to shear and displayed increased modulus values at high frequencies (Huang, Du, Xiao, & Wang, 2017).

The contribution of hydrogen bonding to viscoelasticity of WPI‐FG coacervates (pH 3.4 and *R* = 1:1, w/w) with varied urea concentration was recorded (Figure 7b). Both *G*′ and *G*″ decreased with increased urea concentration. Hydrogen bonds were decreased by urea, leading to diminished coacervate viscoelasticity due to the loosely packed structural properties. Urea effects on WPI‐FG coacervate viscoelasticity were not as prominent as caused by NaCl at the same concentration. This confirmed our previous conclusion based on observations of BSA‐FG coacervates where we determined that electrostatic interaction primarily contributed to coacervate stability while hydrogen bonding played a secondary role (Girard et al., 2002). Fish gelatin‐GA coacervates formed at lower temperatures had higher *G*′ within the frequency range tested. FT‐IR analysis confirmed that fish gelatin formed helical structure at 10°C due to favored hydrogen bonding caused by decreased temperature. Thus, lower temperature facilitated the formation of a sponge‐like porous microstructure of the fish gelatin‐GA coacervates with the water vacuoles entrapped became smaller and more homogenous in size. This results in a more compact coacervate microstructure due to favored non‐Coulombic interactions, such as hydrogen bonding, which can be related to greater elasticity of the coacervate phase (Anvari, Pan, Yoon, & Chung, 2015).

## 4 CONCLUSIONS

Effects of ionic strength and hydrogen bonding on WPI‐FG coacervate formation and coacervate rheological properties were investigated in this study. Increased ionic strength with added NaCl decreased WPI‐FG pH_c_ and pH_φ1_ but increased pH_φ2_. However, pH_max_ was not affected by increasing NaCl concentration to 100 mM. Suppressed biopolymer electrostatic interactions induced by added NaCl were responsible for the narrower pH range of WPI‐FG coacervate stability with reduced WPI‐FG coacervate particle size and number. Urea, a standard denaturant that decreases hydrogen bonding, shifted pH_φ1_, pH_max_, and pH_φ2_ to lower pHs while pH_c_ was constant with urea concentrations up to 100 mM. Structures of WPI‐FG coacervates were loosely packed due to increased ionic strength and decreased hydrogen bonding caused by NaCl and urea addition, respectively, resulting in reduced dynamic viscosity and viscoelasticity.

## CONFLICT OF INTEREST

The authors declare that there are no conflicts of interest regarding the publication of this paper.

## ETHICAL APPROVAL

This study does not involve any human or animal testing.
